# Unraveling the Transcriptional Basis of Temperature-Dependent Pinoxaden Resistance in *Brachypodium hybridum*

**DOI:** 10.3389/fpls.2017.01064

**Published:** 2017-06-21

**Authors:** Maor Matzrafi, Lidor Shaar-Moshe, Baruch Rubin, Zvi Peleg

**Affiliations:** The Robert H. Smith Institute of Plant Sciences and Genetics in Agriculture, The Robert H. Smith Faculty of Agriculture, Food and Environment, The Hebrew University of JerusalemRehovot, Israel

**Keywords:** ACCase inhibitors, climate change, CYP450, glutathione-*S*-transferase, metabolic resistance, reactive oxygen species, RNA-seq, temperature-dependent response

## Abstract

Climate change endangers food security and our ability to feed the ever-increasing human population. Weeds are the most important biotic stress, reducing crop-plant productivity worldwide. Chemical control, the main approach for weed management, can be strongly affected by temperature. Previously, we have shown that temperature-dependent non-target site (NTS) resistance of *Brachypodium hybridum* is due to enhanced detoxification of acetyl-CoA carboxylase inhibitors. Here, we explored the transcriptional basis of this phenomenon. Plants were characterized for the transcriptional response to herbicide application, high-temperature and their combination, in an attempt to uncover the genetic basis of temperature-dependent pinoxaden resistance. Even though most of the variance among treatments was due to pinoxaden application (61%), plants were able to survive pinoxaden application only when grown under high-temperatures. Biological pathways and expression patterns of members of specific gene families, previously shown to be involved in NTS metabolic resistance to different herbicides, were examined. Cytochrome P450, glucosyl transferase and glutathione-*S*-transferase genes were found to be up-regulated in response to pinoxaden application under both control and high-temperature conditions. However, biological pathways related to oxidation and glucose conjugation were found to be significantly enriched only under the combination of pinoxaden application and high-temperature. Analysis of reactive oxygen species (ROS) was conducted at several time points after treatment using a probe detecting H_2_O_2_/peroxides. Comparison of ROS accumulation among treatments revealed a significant reduction in ROS quantities 24 h after pinoxaden application only under high-temperature conditions. These results may indicate significant activity of enzymatic ROS scavengers that can be correlated with the activation of herbicide-resistance mechanisms. This study shows that up-regulation of genes related to metabolic resistance is not sufficient to explain temperature-dependent pinoxaden resistance. We suggest that elevated activity of enzymatic processes at high-temperature may induce rapid and efficient pinoxaden metabolism leading to temperature-dependent herbicide resistance.

## Introduction

Anthropogenic greenhouse gas emissions and climate change pose risks to long-term food security due to their detrimental effects on agriculture productivity (Myers et al., 2017). To feed the 9.6 billion people expected by 2050 (FAOSTAT, 2017) a significant increase in cereal-grain yield will be needed (reviewed by Tester and Langridge, 2010). In the long-term (2030–2050), climatic changes in the Middle East are expected to affect mean temperatures by 1–2°C (Parry et al., 2007; Nelson et al., 2009). However, greater risks to food security may be posed by changes in between-year and within-year variability and the increasing frequency and severity of extreme weather events (Gornall et al., 2010; Lelieveld et al., 2016; Stott, 2016). These environmental changes will affect the development and productivity of both crops and weeds. Weed infestation has already been acknowledged as a major factor causing yield reduction in various crops such as maize (*Zea mays*, Soltani et al., 2016), rice (*Oryza sativa*, Chauhan and Johnson, 2011; Chauhan and Opena, 2012), and hazelnut (*Corylus avellana*, Kaya-Altop et al., 2016).

Since their introduction in 1940, herbicides are the most cost-effective and efficient practice for weed control. In recent years, this method of weed control has become less efficient due to the evolution of herbicide-resistant weeds (Heap, 2017). Herbicide resistance is a consequence of strong selection pressure imposed by repeated application of the same herbicide to a weed population. Herbicide resistance can result from modification of the target site (TS) or via other mechanisms involved in non-target site (NTS) resistance (Rubin, 1991). Mechanisms of TS resistance have been well studied; they involve structural changes at herbicide-binding sites or increased expression of target proteins. NTS resistance can be endowed through reduced absorption (Koger and Reddy, 2005), reduced translocation (Feng et al., 2004) or sequestration (Kleinman and Rubin, 2016). The underlying mechanisms involved in NTS resistance are still not thoroughly understood (Délye, 2013).

Acetyl-CoA carboxylase (ACCase) inhibitors are commonly used to control grass weeds in various crops. In most plants, two isoforms of the ACCase enzyme, heteromeric and homomeric, exist in different cell compartments (i.e., cytosol and plastids; Roesler et al., 1997). In grass species, only the homomeric form of the enzyme is present and ACCase inhibitors function by blocking this form (Sasaki and Nagano, 2004). NTS resistance to ACCase inhibitors is endowed mostly by herbicide detoxification (Kaundun, 2014). The first two crucial phases of detoxification are mediated by members of the cytochrome P450 (CYP450) enzyme family (Manabe et al., 2007; Gaines et al., 2014; Iwakami et al., 2014) and both glutathione-*S*-transferase (GST; Cummins et al., 1999, 2013; Skipsey et al., 2011) and glucosyl transferase (GT; Baerson et al., 2005; Gardin et al., 2015; Xu et al., 2015) play key roles in the conjugation of the herbicide. Different genes from all three families (CYP450, GST, and GT) have been found to be up-regulated in herbicide-resistant grass weed populations such as *Lolium rigidum* (Gaines et al., 2014; Duhoux et al., 2015), *Alopecurus myosuroides* (Gardin et al., 2015), and *Eleusine indica* (An et al., 2014; Chen et al., 2015).

Environmental conditions such as temperature can affect the retention, penetration and movement of herbicides through the plant and can also modify plants growth and development, indirectly affecting herbicide activity within the plant (e.g., Hammerton, 1967; Caseley, 1989; Rubin, 1991; Sundby et al., 1993; Robinson et al., 2015). Temperature may modify the response of plants to herbicides with different modes of action (HRAC, 2017). This phenomenon has been demonstrated in the effect of paraquat (group D) on *Hordeum glaucum* (Lasat et al., 1996), the effect of glyphosate (group G) on *Conyza* sp. (Kleinman et al., 2015), the effect of mesotrione (group F) on *Amaranthus palmeri* (Godar et al., 2015), and the effect of pinoxaden (group A) on *Brachypodium hybridum* (Matzrafi et al., 2016).

*Brachypodium*, a small annual grass species, native to the Mediterranean region, is a valuable model system for a variety of biological processes and genome organization in cereals (reviewed by Kellogg, 2015). In recent years, it has emerged as a powerful model plant for the study of herbicide resistance in grass weeds (e.g., Gressel et al., 1983; Matzrafi et al., 2014; Frenkel et al., 2017). Recently, we demonstrated that elevated temperatures result in increased tolerance to ACCase inhibitors in various grass weed species (Matzrafi et al., 2016). Here, we employed a system biology approach to uncover the transcriptional basis of temperature-dependent NTS resistance mechanism. We hypothesized that temperature-dependent herbicide detoxification is facilitated by enhanced enzymatic efficiency at elevated temperatures. Previously, we have identified a *B. hybridum* accession, presenting temperature-dependent resistant to pinoxaden (Matzrafi et al., 2016). The aims of the current study were to: (i) characterize the transcriptional differences between pinoxaden-treated and untreated plants under different temperatures, (ii) elucidate the biological processes that are associated with temperature-dependent herbicide detoxification, and (iii) examine the role of metabolism-related genes known to be involved in herbicide resistance in temperature-dependent pinoxaden resistance in *B. hybridum*.

## Materials and Methods

### Plant Material and Growth Conditions

Seeds of *B. hybridum* accession BrI-782 (temperature-dependent NTS-resistant to the ACCase inhibitor pinoxaden; Matzrafi et al., 2014) were germinated in trays filled with growth mixture (Pele-Shacham, Israel). The trays were placed in a dark, cold room (16°C) until germination. After emergence, uniform seedlings were transplanted into pots (7 cm × 7 cm × 6 cm) containing similar growth mixture and transferred to a phytotron where they were kept under natural Mediterranean growth conditions [10/16°C (night/day), 10 h of light]. Two temperature regimes were used in this study: control [10/16°C (night/day)] and high temperature [28/34°C (night/day)].

At the three-leaf stage (BBCH scale 13; Hong et al., 2011), plants were treated with either water (control) or the recommended dose of the ACCase inhibitor pinoxaden (Axial^®^, 50 g L^-1^ pinoxaden + 11.25 g L^-1^ cloquintocet-mexyl, EC, Syngenta, Switzerland; X = recommended dose of 30 g ai ha^-1^). The treatment was applied using a chain-driven sprayer delivering 300 L ha^-1^. One hour after treatment (HAT), plants were moved back to the phytotron and each plant was assigned to one of two temperature regimes: control or high. In each room, 10 plants (five treated with pinoxaden and five treated with water) were kept for 21 days after treatment (DAT). Survival rates were visually assessed and shoot fresh weight was measured.

### Sample Preparation and RNA Sequencing

**FIGURE 1:**
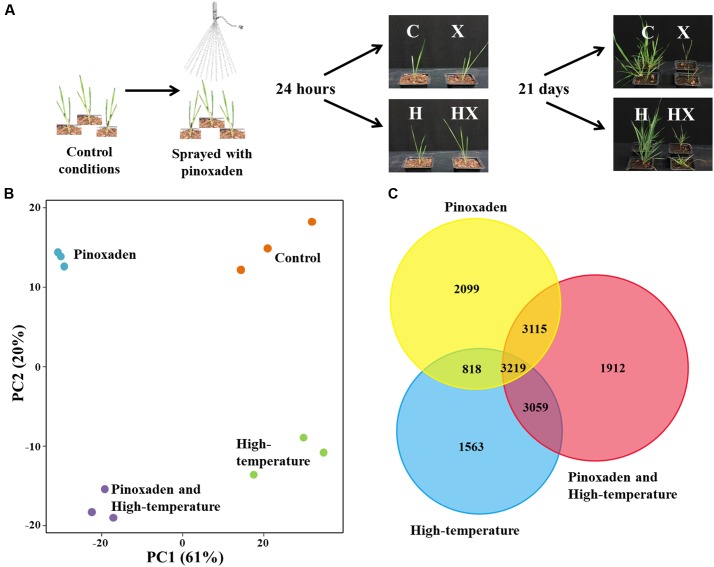
**(A)** Experimental design and final phenotypes of *Brachypodium hybridum* plants (accession BrI-782) grown under control (C, 10/16°C day/night) and high-temperature (H, 28/34°C) conditions. Plants were sprayed with pinoxaden (X) or water at the three-leaf stage; RNA-seq samples were taken 24 h after application. Plant survival rates were determined 21 days after pinoxaden application. **(B)** Principal-component (PC) analysis of rlog transformed gene expression data, generated by RNA-sequencing. Each treatment contains three biological repeats and is indicated by a different color. **(C)** Venn diagram of differentially expressed genes (DEGs) from all three treatments.

Samples of fresh shoot tissue were collected from treated and untreated plants at 24 HAT (**Figure 1A**), immediately frozen in liquid nitrogen and stored at -80°C. RNA-seq analysis was conducted using three plants from each of the following treatments: control (C), pinoxaden application (X), high-temperature (H) and the combination of pinoxaden and high-temperature (HX; **Figure 1A**). Total RNA was extracted using a Plant/Fungi Total RNA Purification Kit (Norgen Biotek Corp., Canada). Total RNA was treated with TURBODNase^®^ (RNase-Free; Ambion, Warrington, United Kingdom) to eliminate DNA contamination. RNA was quantified using a NanoDrop (ND-1000) spectrophotometer (Thermo Scientific, Wilmington, DE, United States) and RNA integrity and quality were assessed with a 2100 Bioanalyzer (Agilent Technologies Inc., Germany). Additional data concerning sample quality and other parameters are presented in Supplementary Table S1.

cDNA libraries were generated using a NEBNext Ultra Directional RNA Prep Kit (New England Biolabs, Ipswich, MA, United States), following the manufacturer’s instructions. After verifying their quality, libraries were indexed with six-nucleotide barcodes and sequencing was performed on the Illumina HiSeq2000 machine using multiplexing for generating 50 base-paired (bp) end reads. Sequencing was carried out at the Technion Genome Center (Haifa, Israel).

### RNA-Sequencing Analysis

*B. hybridum* transcript levels were obtained from HiSeq2000 machine using a custom computational pipeline. Briefly, 50 bp end reads were trimmed and quality-filtered using Trimmomatic (Bolger et al., 2014) and then mapped to the *B. distachyon* genome (International Brachypodium Initiative, 2010), version 3.0 using Tophat (Kim D. et al., 2013). Tophat was run with a maximum intron length of 10,000 bases to reduce the likelihood of false positives (Walters et al., 2013). The mean percentages of overall mapped reads and multiple mapped reads across samples were 80 and 5%, respectively. Mapped reads were then checked for overlap with JGI v3.1^1^ gene exons using htseq-count (Anders et al., 2015). The mean percentage of reads overlapping (a known feature across samples) was 90%. We then used DESeq2 (Love et al., 2014) to detect differentially expressed genes (DEGs) among samples from the different experimental treatments (high-temperature, pinoxaden and their combination) and the control. Data is presented as log2 of fold-change values (log2FC). For DEG analysis we considered genes that were significant at false discovery rate (FDR) ≤ 5%.

### Functional Annotation of Transcriptome Analysis

Differentially expressed genes were analyzed using hierarchical clustering and divided into parallel plots using the JMP (ver. 12) statistical package (SAS Institute Inc., Cary, NC, United States). Genes that were highly up-regulated under HX treatment were annotated with MapMan software (Thimm et al., 2004) using a functional data base containing 32,031 different assigned identifiers, with more than 35 general biological processes matching the *B. distachyon* genome (International Brachypodium Initiative, 2010). Pathway-enrichment analysis was performed with FunRich, a functional enrichment analysis software tool^2^ (FDR ≤ 0.05; Pathan et al., 2015), using MapMan annotation as reference data (Thimm et al., 2004).

### Quantitative PCR

RNA samples used for RNA-seq analysis (*n* = 3) as well as additional three plants that were grown and treated with the plants selected for RNA-seq analysis were used for qPCR validation (*n* = 6). First-strand cDNA was synthesized using qScript^TM^ cDNA Synthesis Kit (Quanta Biosciences Inc., United States), following the manufacturer’s instructions. qPCR was carried out using PerfeCTa^®^ SYBR^®^ Green FastMix^®^ (Quanta Biosciences Inc., United States) in a PikoReal RT-PCR system (Thermo Fisher Scientific Inc., United States). Gene-specific primers were designed using Primer3 software (Supplementary Table S2). PCR mixtures included 2 μL of cDNA (diluted by four), 8 μL of SYBR Mix, and 300 nM of each primer in a final volume of 10 μL. The 2^-ΔΔCT^ method (Livak and Schmittgen, 2001) was used for the normalization and calibration processes. Transcript values were relatively tested compared to the housekeeping gene *S*-adenosylmethionine decarboxylase (SamDC, BRADI2G02580; Hong et al., 2008), whose expression was not affected by the herbicide and∖or high-temperature treatment.

### ROS Staining and Imaging

Plants of *B. hybridum* accession BrI-782 were grown under controlled conditions, as described above. At the three-leaf stage, plants were treated with the recommended dose of pinoxaden as described above. Treated and untreated plants were transferred back to controlled conditions or subjected to a high-temperature regime. The experiment was conducted three times using three replicates for each treatment. ROS measurements were taken at 2, 8, and 24 HAT. Dichlorodihydrofluorescein diacetate (H_2_DCF-DA) was used as a probe for H_2_O_2_/peroxides as described previously (Pena et al., 2012). A 50 μM stock of H_2_DCF-DA was prepared in DMSO, diluted (1:9) in a 10 mM Tris-HCl (pH 7.4) and non-ionic surfactant (Spreader L77, ADAMA-Agan, Israel) at 0.05% v/v was added. The second leaf of each plant was cut and covered with aluminum foil, to prevent light exposure for 30 min, during which time the leaf was kept at room temperature. Leaves were than washed three times with 10 mM Tris-Hcl (pH 7.4). Fluorescent stereoscope images were captured using a Nikon SMZ1500 (Nikon, Japan) zoom stereoscope, with an excitation filter of 450–490 nm and a barrier filter of 510 nm, which was used to detect H_2_DCFDA green fluorescence. Images were captured with a color camera (DS RI1, Nikon, Japan) operated with NIS Elements V3 software (Nikon, Japan). Exposure times were equal for all samples. Autofluorescence was not observed in unstained controls at the exposure time used. Leaf fluorescence was quantified using ImageJ software (ver. 1.63; U.S. National Institutes of Health).

### Accession Number

Raw sequencing files of mRNA sequencing are available at the short read archive of the National Center for Biotechnology Information (https://trace.ncbi.nlm.nih.gov/Traces/sra) under accession number PRJNA360668.

## Results

As a native Mediterranean temperate grass species, *B. hybridum* grows during the winter season (November to April). Under control conditions (10/16°C, night/day), which mimic the Mediterranean winter, BrI-782 plants showed a higher sensitivity to pinoxaden that was manifested by lower shoot fresh weights (21%) and a lower survival rate, as compared with treated plants grown under high-temperature (**Figure 1A** and Supplementary Table S3). We hypothesized that the significant differences in plant response to pinoxaden under contrasting temperature regimes may be the result of temperature-dependent transcriptional modifications. In order to test our hypothesis, we used RNA-seq to detect the temperature-dependent differences in transcripts, pathways and mechanisms between pinoxaden-sensitive and pinoxaden-resistant plants.

### RNA-Seq Data

A total of 257 million raw reads were generated from the 12 libraries (three biological replicates for each of the four treatments). After removing reads containing adaptor or ploy-N and low-quality reads, 208 million clean reads were obtained with 13.4–18.6 million reads per sample. In the absence of a fully sequenced *B. hybridum* genome, the annotation procedure was carried using the *B. distachyon* database. Our *B. hybridum* samples scored 80.6% mapped reads and 87.2% featured contigs (Supplementary Table S1). Principal-component analysis was used to assess the variability among biological replicates and treatments. Within each treatment, the three biological replicates showed a high degree of similarity to one another and formed an independent cluster. Among the treatments, application of herbicide either alone or in combination with high-temperature resulted in a separation of the control and high-temperature treatments. This separation explained 61% of the total experimental variance (**Figure 1B**).

The number of DEGs between each treatment and control conditions ranged between 11,305 for the combination of herbicide and high-temperature, 9,253 for the herbicide application, and 8,659 for the high-temperature treatment (Supplementary Table S4). A search for common DEGs among all treatments yielded 3,219 genes. The combined herbicide and high-temperature treatment was very similar to both the herbicide application and the high-temperature treatment with 3,115 and 3,059 common DEGs, respectively (**Figure 1C**). The similarity of the combination treatment to the other two single treatments correlates with the dual effect of both stress factors together. The combination of herbicide and high-temperature had the highest number of up-regulated DEGs (5,377), as compared to the herbicide application (4,415) and the high-temperature treatment (3,822; Supplementary Table S4).

### Biological Process Analysis

**FIGURE 2:**
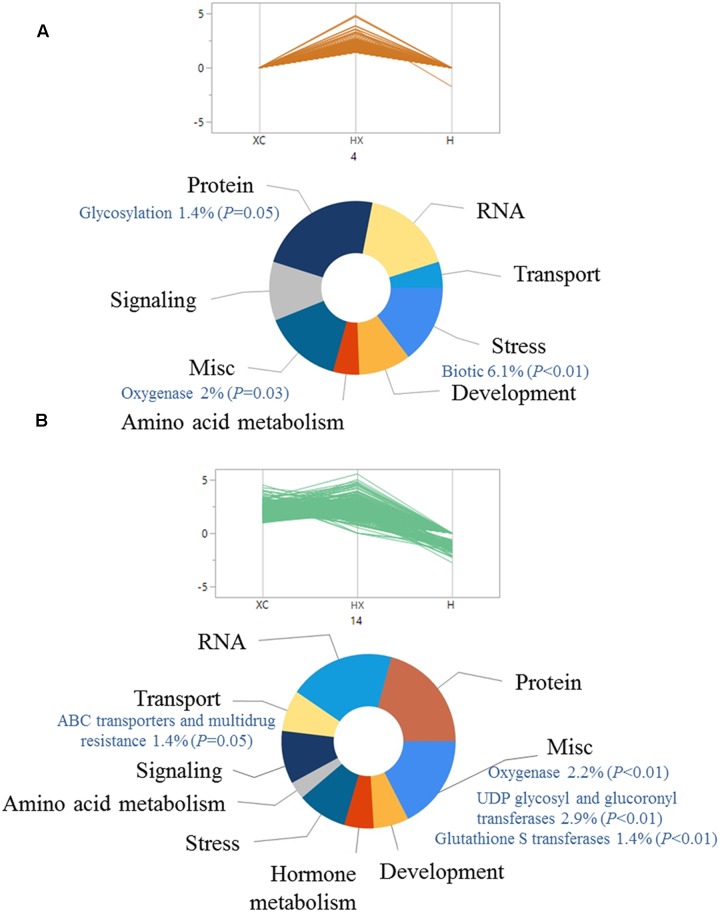
Functional classification of significantly (FDR ≤ 0.05) up-regulated genes in pinoxaden-treated plants under high-temperature regime, compared with the two other treatments (pinoxaden and high-temperature). All genes were divided into 25 groups using hierarchal clustering analysis according to the expression pattern of all three treatments. Two clusters, **(A)** 4 and **(B)** 14, were chosen as representative trends for up-regulated genes. Doughnut charts were produced based on MapMan annotations and the FunRich platform.

The pattern of gene expression was analyzed using hierarchical clustering of 3,129 common DEGs across all treatments. DEGs were classified into 25 groups by parallel plots (Supplementary Table S5), which enabled us to identify specific trends in gene expression. Assuming that over-expression of DEGs can lead to enhanced herbicide detoxification (Délye, 2013), we focused on groups that included DEGs that were up-regulated mainly in the combined treatment (groups 4 and 14 in **Figures 2A,B**, respectively; Supplementary Table S5). Up-regulation of genes from both plots was slightly different, as plot 4 showed greater differences in the combined treatment, as compared with plot 14 (**Figure 2**). Enrichment analysis was conducted to identify key biological processes related to temperature-dependent pinoxaden detoxification. Both groups included genes associated with protein, RNA, stress, and transport (**Figures 2A,B**). Examination of specific pathways within each category showed that both groups were enriched with genes associated with oxygenase activity (*P* = 0.03, *P* < 0.01, respectively) as well as conjugation-related pathways, including genes associated with UDP-glucosyl and glucuronyl transferase (*P* < 0.01) and GST (*P* < 0.01; **Figures 2A,B**). Genes associated with ABC transporters and multidrug resistance, representative of the final stage in herbicide detoxification, were also significantly enriched (*P* = 0.05; **Figure 2B**).

### Candidate Metabolic-Resistance Genes

**FIGURE 3:**
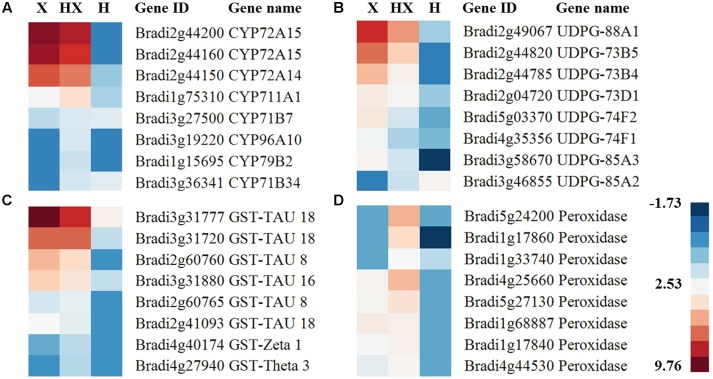
Heatmap of herbicide-associated genes among the different treatments relative to control conditions. X, pinoxaden; H, high-temperature, and HX, combination of high-temperature and pinoxaden. **(A)** Cytochrome P450, **(B)** UDP-glucosyl and glucuronyl transferase, **(C)** glutathione-*S*-transferase, and **(D)** peroxidase. Blue and red colors represent low and high relative expression compared with the mean value of expression across all samples, respectively. Scale is log_2_ of mean expression value.

Herbicide metabolism is generally composed of four different phases, systematically rendering the herbicide molecule into a non-toxic product (Carvalho et al., 2009). We identified DEGs that are involved in these processes (FDR ≤ 0.05) and used qPCR to validate their expression levels under the different treatments (Supplementary Figure S1). Two DEGs, Bradi2g44160 and Bradi2g44200, were annotated as members of the CYP72A subfamily, which are associated with the first phase of herbicide metabolism. Genes of this subfamily were recently reported to be up-regulated in *L. rigidum* plants resistant to an ACCase inhibitor (Gaines et al., 2014). In our study, these genes were highly up-regulated (log_2_FC > 7.9) under both the herbicide application and the combination of herbicide and high-temperature treatments (**Figure 3A** and Supplementary Table S6). Other genes involved in CYP450 catalytic activity encode for NADP enzymes (Davydov, 2001), which were also found to play a role in the process of ROS quenching (Buchanan et al., 2006). NAD(P)-linked oxidoreductase (Bradi3g48197) was up-regulated with log_2_FC values of 3.3 and 4.6 under high-temperature and combination of herbicide and high-temperature treatments, respectively (Supplementary Table S5). Additional CYP450 genes, CYP96A10 (Bradi3g19220) and CYP79B2 (Bradi1g15695), were up-regulated only in the combination of herbicide and high-temperature, but at lower levels than the CYP72A genes (log_2_FC < 2.6; **Figure 3A** and Supplementary Table S6).

With regard to the second phase of herbicide metabolism, DEGs involved in GT processes, UDP-glucosyl and glycosyl transferase (UDPGT); UDPGT88A1 (Bradi2g49067), UDPGT73B5 (Bradi2g44820), and UDPGT85A2 (Bradi3g46855) were up-regulated under herbicide application and the combination of herbicide and high-temperature (**Figure 3B** and Supplementary Table S7). Another major group of second-phase-related genes, GSTs, were also examined. GST DEGs (Bradi3g31777, Bradi3g61100 and others) were up-regulated in response to both herbicide treatments (herbicide application and combination of herbicide and high-temperature; **Figure 3C** and Supplementary Table S8). The trends observed in the RNA-seq experiment for these genes were also observed in the qPCR analysis (Supplementary Figure S1).

### ROS Accumulation in Response to Different Treatments

CYP450 activity, which produces ROS, is also regulated by the accumulation of ROS (Davydov, 2001; Narusaka et al., 2004; McIntosh et al., 2014). Peroxidases are known to act as part of ROS neutralization mechanisms (Meunier and Bernadou, 2000). A high expression of two DEGs (Bradi5g24200 and Bradi4g25660), annotated as peroxidases, was detected only under the combination of herbicide and high-temperature (log_2_FC > 3.5; **Figure 3D** and Supplementary Table S9). The multiprotein bridging factor 1C (MBF) was previously described as a key regulatory element linking ROS signaling to stress responses (Miller et al., 2008). MBF (Bradi1g37080) showed a higher expression level under the combination of herbicide and high-temperature, as compared with the other treatments (herbicide application and high-temperature treatments) with log_2_FC values of 8.1, 6.6 and 7.4, respectively (Supplementary Table S5). This trend can be correlated with greater activity of CYP450 enzymes, leading to more ROS neutralization, as part of the herbicide-resistance mechanism.

**FIGURE 4:**
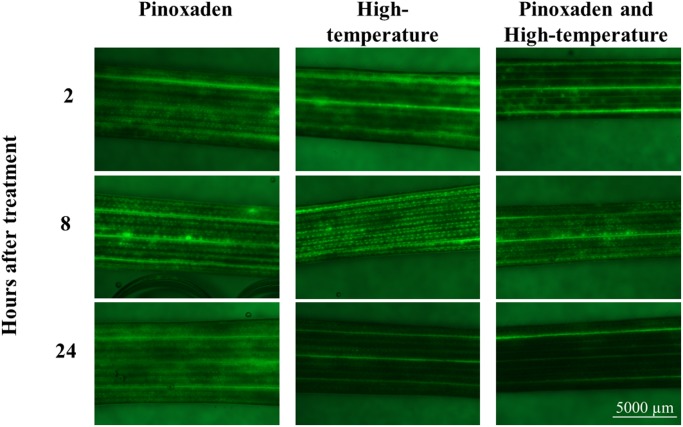
Visual presentation of the accumulation of reactive oxygen species using H_2_DCF-DA dye. Plants were treated with: pinoxaden, high-temperature (28/34°C) and high-temperature and pinoxaden application. Photos were taken 2, 8, and 24 h after herbicide application.

To learn more about the role of ROS in the herbicide-resistance mechanism, ROS staining was conducted and the patterns of ROS accumulation among the different treatments were examined. ROS quantities did not differ significantly among the different treatments at 2 and 8 HAT. However, at 24 HAT, plants subjected to the combination of herbicide and high-temperature had ROS levels that were significantly lower than those detected under control conditions (**Figure 4** and Supplementary Table S10).

## Discussion

Elevated temperatures have been shown to have a direct effect on the level of NTS herbicide resistance in various weed species (Godar et al., 2015; Kleinman et al., 2015; Matzrafi et al., 2016). Here, we describe for the first time the transcriptional basis of a temperature-dependent NTS herbicide-resistance mechanism in *B. hybridum* and reveal the importance of temperature in herbicide-related metabolic processes. Biochemical analysis of temperature-dependent pinoxaden detoxification suggests two main stages of oxidation and glucose conjugation as key steps in the resistance mechanism (Matzrafi et al., 2016). Exploring transcriptional-enriched pathways that were differentially regulated under the combination of herbicide and high-temperature revealed up-regulation of several known herbicide defense-related genes (e.g., oxygenases, GST, GT, and ABC transporters; **Figures 2A,B**). The enrichment of these pathways reinforces previous findings and indicates a higher rate of pinoxaden metabolism at high-temperature. Members of the CYP72A subfamily that were previously identified as components of the mechanism of resistance to ACCase and acetolactate synthase inhibitors (Gaines et al., 2014; Saika et al., 2014) were also found to be highly expressed in pinoxaden-treated plants under both temperature regimes (**Figure 3A**). The abundance of genes from the CYP72A subfamily identified in different studies involving different inhibitors and weed species (Imaishi and Matumoto, 2007; Gaines et al., 2014; Iwakami et al., 2014; Saika et al., 2014) supports the assumption of their role in this mechanism of herbicide resistance. Previous studies suggested the involvement of GT activity in resistance to ACCase inhibitors (Menendez and De Prado, 1996; Brazier et al., 2002), which function mainly at the second phase of herbicide detoxification (McFadden et al., 1989; Baerson et al., 2005). Expression levels of different transcripts encoding CYP72A and UDPGT genes that were detected in our work can be associated with the formation of specific pinoxaden metabolites previously found in *B. hybridum* plants (Matzrafi et al., 2016). The detection of these DEGs following pinoxaden application in both temperature treatments (herbicide application and the combination of herbicide and high-temperature) suggests that the observed differences in plant response to pinoxaden are not solely due to the up-regulation of these genes. Further investigation of the role of the 2,099 DEGs that were found to be expressed only under pinoxaden treatment is needed to help to shed light on their role in herbicide response.

The catalytic activity of CYP450 enzymes includes the splitting of O_2_ molecules to form a hydroxylated product (Munro et al., 2013). In this process, ROS are formed and transformed into H_2_O (Davydov, 2001; McIntosh et al., 2014). Both NADP and peroxidases were previously suggested to play a role in CYP450 catalytic activity and the quenching of ROS (Davydov, 2001; Buchanan et al., 2006). The up-regulation of NADP and peroxidases that was unique to the combination of herbicide and high-temperature treatment can be correlated with the rapid metabolism of pinoxaden leading to herbicide resistance. The direct damage caused by ROS following the application of herbicides that function as photosystem I and II inhibitors (Fujii et al., 1990; Fuerst and Norman, 1991) is well known. In studies of other types of herbicides, this phenomenon is less familiar, but over time, as in almost any other stress response, ROS will form. The significant reduction in ROS content 24 HAT under the combination of herbicide and high-temperature, as compared with all other treatments, may indicate more efficient metabolic activity of ROS scavengers at high-temperature.

Recent studies have suggested a new approach for understanding ROS activity, as ROS have been found to act as beneficial elements in the context of many physiological functions, such as programmed cell death and cellular proliferation (Mittler, 2016). In previous studies, ROS were shown to have an effect on the expression pattern of CYP450 enzymes, inducing the expression of CYP81D8 in *Arabidopsis* (Desikan et al., 2001). Over-expression of additional plant defense-related genes such as GSTs [involved in antioxidant stress, lipid peroxidation (Cummins et al., 2013), detoxification of ACCase inhibitors (Edwards and Cole, 1996; Cummins et al., 1999)], and MBF, which is involved in transcriptional co-activation of ROS signaling (Miller et al., 2008), were also detected the combination of herbicide and high-temperature treatment. Herbicide metabolism processes can also be associated with several chaperoning components related to regulatory elements and activating genes. Several HSP related DEGs, mainly HSP20 (Bradi3g58590 and Bradi1g44230), were highly expressed in response to high-temperature and combination of herbicide and high-temperature (Supplementary Table S5). It can be hypothesized that this high expression is part of a general stress response (Waters, 2013), yet variation in the level of expression can suggest different activation rate of downstream resistance related components. High expression of regulatory element such as NAC related genes (Bradi1g63600 and Bradi4g44000), involved in response to biotic and abiotic stress responses (Puranik et al., 2012), was also found. Activators such as protein kinase (Bradi1g65930) and Calcium-binding (Bradi1g53830 and Bradi4g30100) related DEGs (Supplementary Table S5) were highly expressed under herbicide application. These genes can be correlated to the function of peroxidases and other enzymes related to herbicide detoxification processes (Kim Y.S. et al., 2013; Alberto et al., 2016). Our results suggest an orchestrated regulation, at the transcriptional and biochemical levels that facilitates temperature-dependent pinoxaden resistance, which is activated upon application of pinoxaden at high-temperature. These factors encourage a rapid and efficient pinoxaden detoxification that eventually results in plant survival.

Monooxygenation activity of CYP72A genes has been widely discussed in the context of herbicide resistance (Prall et al., 2016). However, only plants subjected to high-temperature survived pinoxaden treatment. Thus, the expression of CYP450 genes may not be related to their monooxygenation activity. On the other hand, temperature has been shown to play a key role in the enzymatic efficiency of CYP450 (Puntarulo and Cederbaum, 1989; Puchkaev et al., 2002). These findings help to explain the differences in plant survival between herbicide application and the combination of herbicide and high-temperature, as pinoxaden was rapidly metabolized only under high-temperature.

## Conclusion

Our previous (Matzrafi et al., 2016) and current results on temperature-dependent herbicide resistance have emphasized the evolutionary effect of climate change on herbicide resistance and, by extension, global agriculture. We propose that temperature-dependent pinoxaden resistance may be affected more by enzymatic efficiency than by gene regulation. It is suggested that the over-expression of metabolic-related genes (CYP72A, GST and GT) is not sufficient for effective herbicide metabolism, as was previously suggested. While previous studies, quantified levels of gene expression based on comparisons between sensitive and resistant populations (i.e., different genetic background), here we used sensitive and resistant plants with the same genetic background (Accession BrI-782). It could be argued that the differences found in the current study were not detected in previous studies, due to the differences in genetic backgrounds. Thus, our strategy enabled to improve our understanding on NTS resistance mechanism under climate change. Exploring the mechanism of temperature-dependent resistance in various weed species may reveal further hub genes and up-stream regulators that affect NTS resistance.

## Author Contributions

MM designed and conducted experiments. MM and LS-M analyzed the data and drafted the paper. BR and ZP designed experiments and wrote the paper. All authors read and approved the manuscript.

## Conflict of Interest Statement

The authors declare that the research was conducted in the absence of any commercial or financial relationships that could be construed as a potential conflict of interest.

## Footnotes

1 http://genome.jgi.doe.gov

2 http://www.funrich.org

## References

[B1] Alberto D. Serra A.-A. Sulmon C. Gouesbet G. Couée I. (2016). Herbicide-related signaling in plants reveals novel insights for herbicide use strategies, environmental risk assessment and global change assessment challenges. *Sci. Total Environ.* 56 1618–1628. 10.1016/j.scitotenv.2016.06.064 27318518

[B2] An J. Shen X. Ma Q. Yang C. Liu S. Chen Y. (2014). Transcriptome profiling to discover putative genes associated with paraquat resistance in goosegrass (*Eleusine indica* L.). *PLoS ONE* 9:99940 10.1371/journal.pone.0099940 PMC4057336 24927422

[B3] Anders S. Pyl P. T. Huber W. (2015). HTSeq-A python framework to work with high-throughput sequencing data. *Bioinformatics* 31 166–169. 10.1093/bioinformatics/btu638 25260700 PMC4287950

[B4] Baerson S. R. Sánchez-Moreiras A. Pedrol-Bonjoch N. Schulz M. Kagan I. A. Agarwal A. K. (2005). Detoxification and transcriptome response in *Arabidopsis* seedlings exposed to the allelochemical benzoxazolin-2(3H)-one. *J. Biol. Chem.* 280 21867–21881. 10.1074/jbc.M500694200 15824099

[B5] Bolger A. M. Lohse M. Usadel B. (2014). Trimmomatic: a flexible trimmer for Illumina sequence data. *Bioinformatics* 30 2114–2120. 10.1093/bioinformatics/btu170 24695404 PMC4103590

[B6] Brazier M. Cole D. J. Edwards R. (2002). O-Glucosyltransferase activities toward phenolic natural products and xenobiotics in wheat and herbicide-resistant and herbicide-susceptible black-grass (*Alopecurus myosuroides*). *Phytochemistry* 59 149–156. 10.1016/S0031-9422(01)00458-7 11809449

[B7] Buchanan B. B. Gruissem W. Jones R. (2006). *Biochemistry and Molecular Biology of Plants.* Rockville, MD: American Society of Plant Physiologists, 588–589.

[B8] Carvalho S. J. De Prado R. Nicolai M. Ferreira R. R. DeOliveria Figueira A. V. Christoffoleti P. J. (2009). Herbicide selectivity by differential metabolism: considerations for reducing crop damages. *Sci. Agric.* 66 136–142. 10.1590/S0103-90162009000100020

[B9] Caseley J. C. (1989). Variation in foliar pesticide performance attributable to humidity, dew and rain effects. *Asp. Appl. Biol.* 21 1215–1225.

[B10] Chauhan B. S. Johnson D. E. (2011). Growth response of direct-seeded rice to oxadiazon and bispyribac-sodium in aerobic and saturated Soils. *Weed Sci.* 59 119–122. 10.1614/WS-D-10-00075.1

[B11] Chauhan B. S. Opena J. (2012). Effect of tillage systems and herbicides on weed emergence, weed growth, and grain yield in dry-seeded rice systems. *Field Crop Res.* 137 56–69. 10.1016/j.fcr.2012.08.016

[B12] Chen S. McElroy J. S. Dane F. Peatman E. (2015). Optimizing transcriptome assemblies for *Eleusine indica* leaf and seedling by combining multiple assemblies from three de novo assemblers. *Plant Genome* 8 10.3835/plantgenome2014.10.0064 33228277

[B13] Cummins I. Cole D. J. Edwards R. (1999). A role for glutathione transferases functioning as glutathione peroxidases in resistance to multiple herbicides in black-grass. *Plant J.* 18 285–292. 10.1046/j.1365-313X.1999.00452.x 10377994

[B14] Cummins I. Wortley D. J. Sabbadin F. He Z. Coxon C. R. Straker H. E. (2013). Key role for a glutathione transferase in multiple-herbicide resistance in grass weeds. *Proc. Natl. Acad. Sci. U.S.A.* 110 5812–5817. 10.1073/pnas.1221179110 23530204 PMC3625300

[B15] Davydov D. R. (2001). Microsomal monooxygenase in apoptosis: another target for cytochrome c signaling? *Trends Biochem. Sci.* 26 155–160. 10.1016/S0968-0004(00)01749-7 11246020

[B16] Délye C. (2013). Unravelling the genetic bases of non-target-site-based resistance (NTSR) to herbicides: a major challenge for weed science in the forthcoming decade. *Pest Manag. Sci.* 69 176–187. 10.1002/ps.3318 22614948

[B17] Desikan R. A-H-Mackerness S. Hancock J. T. Neill S. J. (2001). Regulation of the *Arabidopsis* transcriptome by oxidative stress. *Plant Physiol.* 127 159–172. 10.1104/pp.127.1.159 11553744 PMC117972

[B18] Duhoux A. Carrère S. Gouzy J. Delye C. (2015). RNA-Seq analysis of rye-grass transcriptomic response to an herbicide inhibiting acetolactate-synthase identifies transcripts linked to non-target-site-based resistance. *Plant Mol. Biol.* 87 473–487. 10.1007/s11103-015-0292-3 25636204

[B19] Edwards R. Cole D. J. (1996). Glutathione transferases in wheat (*Triticum*) species with activity toward fenoxaprop-ethyl and other herbicides. *Pestic. Biochem. Physiol.* 54 96–104. 10.1006/pest.1996.0013

[B20] FAOSTAT (2017) *Food and Agriculture Organization of the United Nations-Statistics Division* Available at: http://www.fao.org/wsfs/world-summit/en

[B21] Feng P. C. C. Tran M. Chiu T. Douglas Sammons R. Heck G. R. Jacob C. A. (2004). Investigations into glyphosate-resistant horseweed (*Conyza canadensis*): retention, uptake, translocation, and metabolism. *Weed Sci.* 52 498–505. 10.1614/WS-03-137R

[B22] Frenkel E. Matzrafi M. Rubin B. Peleg Z. (2017). Effects of environmental conditions on the fitness penalty in herbicide resistant *Brachypodium hybridum*. *Front. Plant Sci.* 8:94 10.3389/fpls.2017.00094 PMC5289963 28217132

[B23] Fuerst E. P. Norman M. A. (1991). Interactions of herbicides with photosynthetic electron transport. *Weed Sci.* 39 458–464.

[B24] Fujii T. Yokoyama E. Inoue K. Sakurai H. (1990). The sites of electron donation of photosystem I to methyl viologen. *Biochim. Biophys. Acta - Bioenerg.* 1015 41–48. 10.1016/0005-2728(90)90213-N

[B25] Gaines T. A. Lorentz L. Figge A. Herrmann J. Maiwald F. Ott M.-C. (2014). RNA-Seq transcriptome analysis to identify genes involved in metabolism-based diclofop resistance in *Lolium rigidum*. *Plant J.* 78 865–876. 10.1111/tpj.12514 24654891

[B26] Gardin J. A. C. Gouzy J. Carrère S. Délye C. (2015). ALOMYbase, a resource to investigate non-target-site-based resistance to herbicides inhibiting acetolactate-synthase (ALS) in the major grass weed *Alopecurus myosuroides* (black-grass). *BMC Genomics* 16:590 10.1186/s12864-015-1804-x PMC4534104 26265378

[B27] Godar A. S. Varanasi V. K. Nakka S. Prasad P. V. V. Thompson C. R. Mithila J. (2015). Physiological and molecular mechanisms of differential sensitivity of Palmer amaranth (*Amaranthus palmeri*) to mesotrione at varying growth temperatures. *PLoS ONE* 10:0126731 10.1371/journal.pone.0126731 PMC4437998 25992558

[B28] Gornall J. Betts R. Burke E. Clark R. Camp J. Willett K. (2010). Implications of climate change for agricultural productivity in the early twenty-first century. *Philos. Trans. R. Soc. B Biol. Sci.* 365 2973–2989. 10.1098/rstb.2010.0158 PMC2935125 20713397

[B29] Gressel J. Regev Y. Malkin S. Kleifeld Y. (1983). Characterization of an s- triazine-resistant biotype of *Brachypodium distachyon*. *Weed Sci.* 31 450–456.

[B30] Hammerton J. L. (1967). Environmental factors and susceptibility to herbicides. *Weed Sci.* 15 330–336. 10.2307/4041001

[B31] Heap I. (2017). *The International Survey of Herbicide-Resistant Weeds.* Available at: http://www.weedscience.com

[B32] Hong S.-Y. Park J.-H. Cho S.-H. Yang M.-S. Park C.-M. (2011). Phenological growth stages of *Brachypodium distachyon*: codification and description. *Weed Res.* 51 612–620. 10.1111/j.1365-3180.2011.00877.x

[B33] Hong S.-Y. Seo P. J. Yang M.-S. Xiang F. Park C.-M. (2008). Exploring valid reference genes for gene expression studies in *Brachypodium distachyon* by real-time PCR. *BMC Plant Biol.* 8:112–123. 10.1186/1471-2229-8-112 18992143 PMC2588586

[B34] HRAC (2017). *Report of the Herbicide Resistance Action Committee.* Available at: http://www.hracglobal.com

[B35] Imaishi H. Matumoto S. (2007). Isolation and functional characterization in yeast of CYP72A18 a rice cytochrome P450 that catalyzes (ω-1)-hydroxylation of the herbicide pelargonic acid. *Pestic. Biochem. Physiol.* 88 71–77. 10.1016/j.pestbp.2006.09.003

[B36] International Brachypodium Initiative (2010). Genome sequencing and analysis of the model grass *Brachypodium distachyon*. *Nature* 463 763–768. 10.1038/nature08747 20148030

[B37] Iwakami S. Uchino A. Kataoka Y. Shibaike H. Watanabe H. Inamura T. (2014). Cytochrome P450 genes induced by bispyribac-sodium treatment in a multiple-herbicide-resistant biotype of *Echinochloa phyllopogon*. *Pest Manag. Sci.* 70 549–558. 10.1002/ps.3572 23650123

[B38] Kaundun S. S. (2014). Resistance to acetyl-CoA carboxylase-inhibiting herbicides. *Pest Manag. Sci.* 70 1405–1417. 10.1002/ps.3790 24700409

[B39] Kaya-Altop E. Haghnama K. Sariaslan D. Phillippo C. J. Mennan H. Zandstra B. H. (2016). Long-term perennial weed control strategies: economic analyses and yield effect in hazelnut (*Corylus avellana*). *Crop Prot.* 80 7–14. 10.1016/j.cropro.2015.10.022

[B40] Kellogg E. A. (2015). *Brachypodium distachyon* as a genetic model system. *Annu. Rev. Genet.* 49 1–20. 10.1146/annurev-genet-112414-055135 26393966

[B41] Kim D. Pertea G. Trapnell C. Pimentel H. Kelley R. Salzberg S. L. (2013). TopHat2: accurate alignment of transcriptomes in the presence of insertions, deletions and gene fusions. *Genome Biol.* 14:R36 10.1186/gb-2013-14-4-r36 PMC4053844 23618408

[B42] Kim Y. S. Jung H. Zerin T. Song H. Y. (2013). Protein profiling of paraquat-exposed rat lungs following treatment with Acai (*Euterpe oleracea* Mart.) berry extract. *Mol. Med. Rep.* 7 881–886. 10.3892/mmr.2013.1259 23291665

[B43] Kleinman Z. Ben-Ami G. Rubin B. (2015). From sensitivity to resistance - factors affecting the response of *Conyza* spp. to glyphosate. *Pest Manag. Sci.* 72 1681–1688. 10.1002/ps.4187 26573966

[B44] Kleinman Z. Rubin B. (2016). Non-target-site glyphosate resistance in *Conyza bonariensis* is based on modified subcellular distribution of the herbicide. *Pest Manag. Sci.* 73 246–253. 10.1002/ps.4293 27098558

[B45] Koger K. Reddy C. (2005). Role of absorption and translocation in the mechanism of glyphosate resistance in horseweed (*Conyza canadensis*). *Weed Sci.* 53 84–89. 10.1614/WS-04-102R

[B46] Lasat M. M. DiTomaso J. M. Hart J. J. Kochian L. V. (1996). Resistance to paraquat in *Hordeum glaucum* is temperature dependent and not associated with enhanced apoplasmic binding. *Weed Res.* 36 303–309. 10.1111/j.1365-3180.1996.tb01660.x

[B47] Lelieveld J. Proestos Y. Hadjinicolaou P. Tanarhte M. Tyrlis E. Zittis G. (2016). Strongly increasing heat extremes in the Middle East and North Africa (MENA) in the 21^st^ century. *Clim. Change* 137 245–260. 10.1007/s10584-016-1665-6

[B48] Livak K. J. Schmittgen T. D. (2001). Analysis of relative gene expression data using real-time quantitative PCR and the 2^-ΔΔC_T_^ method. *Methods* 25 402–408. 10.1006/meth.2001.1262 11846609

[B49] Love M. I. Huber W. Anders S. (2014). Moderated estimation of fold change and dispersion for RNA-seq data with DESeq2. *Genome Biol.* 15:550 10.1186/s13059-014-0550-8 PMC4302049 25516281

[B50] Manabe Y. Tinker N. Colville A. Miki B. (2007). CSR1 the sole target of imidazolinone herbicide in *Arabidopsis thaliana*. *Plant Cell Physiol.* 48 1340–1358. 10.1093/pcp/pcm105 17693453

[B51] Matzrafi M. Gadri Y. Frenkel E. Rubin B. Peleg Z. (2014). Evolution of herbicide resistance mechanisms in grass weeds. *Plant Sci.* 229 43–52. 10.1016/j.plantsci.2014.08.013 25443832

[B52] Matzrafi M. Seiwert B. Reemtsma T. Rubin B. Peleg Z. (2016). Climate change increases the risk of herbicide-resistant weeds due to enhanced detoxification. *Planta* 244 1217–1227. 10.1007/s00425-016-2577-4 27507240

[B53] McFadden J. J. Frear D. S. Mansager E. R. (1989). Aryl hydroxylation of diclofop by a cytochrome P450 dependent monooxygenase from wheat. *Pestic. Biochem. Physiol.* 34 92–100. 10.1016/0048-3575(89)90145-4

[B54] McIntosh J. A. Farwell C. C. Arnold F. H. (2014). Expanding P450 catalytic reaction space through evolution and engineering. *Curr. Opin. Chem. Biol.* 19 126–134. 10.1016/j.cbpa.2014.02.001 24658056 PMC4008644

[B55] Menendez J. De Prado R. (1996). Diclofop-methyl cross-resistance in a chlorotoluron-resistant biotype of *Alopecurus myosuroides*. *Pestic. Biochem. Physiol.* 56 123–133. 10.1006/pest.1996.0066

[B56] Meunier B. Bernadou J. (2000). Active iron-oxo and iron-peroxo species in cytochromes P450 and peroxidases; oxo-hydroxo tautomerism with water-soluble metalloporphyrins∖rmetal-oxo and metal-peroxo species in catalytic oxidations. *Struct. Bond.* 97 1–35. 10.1007/3-540-46592-8_1

[B57] Miller G. Shulaev V. Mittler R. (2008). Reactive oxygen signaling and abiotic stress. *Physiol. Plant.* 133 481–489. 10.1111/j.1399-3054.2008.01090.x 18346071

[B58] Mittler R. (2016). ROS are good. *Trends Plant Sci.* 22 11–19. 10.1016/j.tplants.2016.08.002 27666517

[B59] Munro A. W. Girvan H. M. Mason A. E. Dunford A. J. Mclean K. J. (2013). What makes a P450 tick? *Trends Biochem. Sci.* 38 140–150. 10.1016/j.tibs.2012.11.006 23356956

[B60] Myers S. S. Smith M. R. Guth S. Golden C. D. Vaitla B. Mueller N. D. (2017). Climate change and global food systems: potential impacts on food security and undernutrition. *Annu. Rev. Public Health* 38 259–277. 10.1146/annurev-publhealth-031816-044356 28125383

[B61] Narusaka Y. Narusaka M. Seki M. Umezawa T. Ishida J. Nakajima M. (2004). Crosstalk in the responses to abiotic and biotic stresses in *Arabidopsis*: analysis of gene expression in cytochrome P450 gene superfamily by cDNA microarray. *Plant Mol. Biol* 55 327–342. 10.1007/s11103-004-0685-1 15604685

[B62] Nelson G. C. Rosegrant M. W. Koo J. Robertson R. Sulser T. (2009). *Climate Change. Impact on Agriculture and Costs of Adaptation.* Washington, DC: International Food Policy Research Institute.

[B63] Parry M. L. Canziani O. F. Palutikof J. P. van der Linden P. J. Hanson C. E. (eds) (2007). “Climate change 2007: impacts, adaptation and bulnerability,” in *Contribution of Working Group II to the Fourth Assessment Report of the Intergovernmental Panel on Climate Change (IPCC)*, (Cambridge: Cambridge University Press).

[B64] Pathan M. Keerthikumar S. Ang C. S. Gangoda L. Quek C. Y. J. Williamson N. A. (2015). FunRich: an open access standalone functional enrichment and interaction network analysis tool. *Proteomics* 15 2597–2601. 10.1002/pmic.201400515 25921073

[B65] Pena L. B. Barcia R. A. Azpilicueta C. E. Méndez A. A. E. Gallego S. M. (2012). Oxidative post translational modifications of proteins related to cell cycle are involved in cadmium toxicity in wheat seedlings. *Plant Sci.* 196 1–7. 10.1016/j.plantsci.2012.07.008 23017894

[B66] Prall W. Hendy O. Thornton L. E. (2016). Utility of a phylogenetic perspective in structural analysis of CYP72A enzymes from flowering plants. *PLoS ONE* 11:0163024 10.1371/journal.pone.0163024 PMC5036807 27669508

[B67] Puchkaev A. V. Wakagi T. Ortiz deMontellano P. R. (2002). CYP119 plus a *Sulfolobus tokodaii* strain 7 ferredoxin and 2-oxoacid: ferredoxin oxidoreductase constitute a high-temperature cytochrome P450 catalytic system. *J. Am. Chem. Soc.* 124 12682–12683. 10.1021/ja0282036 12392414

[B68] Puntarulo S. Cederbaum A. I. (1989). Temperature dependence of the microsomal oxidation of ethanol by cytochrome P450 and hydroxyl radical-dependent reactions. *Arch. Biochem. Biophys.* 269 569–575. 10.1016/0003-9861(89)90142-2 2537602

[B69] Puranik S. Sahu P. P. Srivastava P. S. Prasad M. (2012). NAC proteins: regulation and role in stress tolerance. *Trends Plant Sci.* 17 369–381. 10.1016/j.tplants.2012.02.004 22445067

[B70] Robinson M. A. Letarte J. Cowbrough M. J. Sikkema P. H. Tardif F. J. (2015). Winter wheat (*Triticum aestivum* L.) response to herbicides as affected by application timing and temperature. *Can. J. Plant Sci.* 95 325–333. 10.4141/cjps-2014-109

[B71] Roesler K. Shintani D. Savage L. Boddupalli S. Ohlrogge J. (1997). Targeting of the *Arabidopsis* homomeric acetyl-coenzyme A carboxylase to plastids of rapeseeds. *Plant Physiol.* 113 75–81. 10.1104/pp.113.1.75 9008389 PMC158117

[B72] Rubin B. (1991). “Herbicide resistance in weeds and crops, prospects progress and,” in *Herbicide Resistance in Weeds and Crops*, eds Caseley J. C. Cussans G. W. Atkin R. K. (Oxford: Butterworth-Heinemann), 387–414. 10.1016/B978-0-7506-1101-5.50001-2

[B73] Saika H. Horita J. Taguchi-Shiobara F. Nonaka S. Ayako N.-Y. Iwakami S. (2014). A novel rice cytochrome P450 gene, CYP72A31 confers tolerance to acetolactate synthase-inhibiting herbicides in rice and *Arabidopsis*. *Plant Physiol.* 166 1232–1240. 10.1104/pp.113.231266 24406793 PMC4226355

[B74] Sasaki Y. Nagano Y. (2004). Plant acetyl-CoA carboxylase: structure, biosynthesis, regulation, and gene manipulation for plant breeding. *Biosci. Biotechnol. Biochem.* 68 1175–1184. 10.1271/bbb.68.1175 15215578

[B75] Skipsey M. Knight K. M. Brazier-Hicks M. Dixon D. P. Steel P. G. Edwards R. (2011). Xenobiotic responsiveness of *Arabidopsis thaliana* to a chemical series derived from a herbicide safener. *J. Biol. Chem.* 286 32268–32276. 10.1074/jbc.M111.252726 21778235 PMC3173150

[B76] Soltani N. Dille A. J. Burke I. C. Everman W. J. VanGessel M. J. Davis V. M. (2016). Potential corn yield losses due to weeds in North America. *Weed Technol.* 30 979–984. 10.1614/WT-D-16-00046.1

[B77] Stott P. (2016). How climate change affects extreme weather events. *Science* 352 1517–1518. 10.1126/science.aaf7271 27339968

[B78] Sundby C. Chow W. S. Anderson J. M. (1993). Effects on photosystem II function, photoinhibition, and plant performance of the spontaneous mutation of serine-264 in the photosystem II reaction center D1 protein in triazine-resistant *Brassica napus* L. *Plant Physiol.* 103 105–113. 10.1104/pp.103.1.105 12231917 PMC158952

[B79] Tester M. Langridge P. (2010). Breeding technologies to increase crop production in a changing world. *Science* 327 818–822. 10.1126/science.1183700 20150489

[B80] Thimm O. Bläsing O. Gibon Y. Nagel A. Meyer S. Krüger P. (2004). MAPMAN: a user-driven tool to display genomics data sets onto diagrams of metabolic pathways and other biological processes. *Plant J.* 37 914–939. 10.1111/j.1365-313X.2004.02016.x 14996223

[B81] Walters B. Lum G. Sablok G. Min X. J. (2013). Genome-wide landscape of alternative splicing events in *Brachypodium distachyon*. *DNA Res.* 20 163–171. 10.1093/dnares/dss041 23297300 PMC3628446

[B82] Waters E. R. (2013). The evolution, function, structure, and expression of the plant sHSPs. *J. Exp. Bot.* 64 391–403. 10.1093/jxb/ers355 23255280

[B83] Xu W. Di C. Zhou S. Liu J. Li L. Liu F. (2015). Rice transcriptome analysis to identify possible herbicide quinclorac etoxification genes. *Front. Genet.* 6:306 10.3389/fgene.2015.00306 PMC4586585 26483837

