# Supplementary material for: Unraveling the Transcriptional Basis of Temperature-Dependent Pinoxaden Resistance in Brachypodium hybridum
Source: Front Plant Sci. 2017 Jun 21;8:1064. doi: 10.3389/fpls.2017.01064 (PMC5478685; doi:10.3389/fpls.2017.01064)
Supplement: Supplementary file 2 [file Presentation_1.PDF]

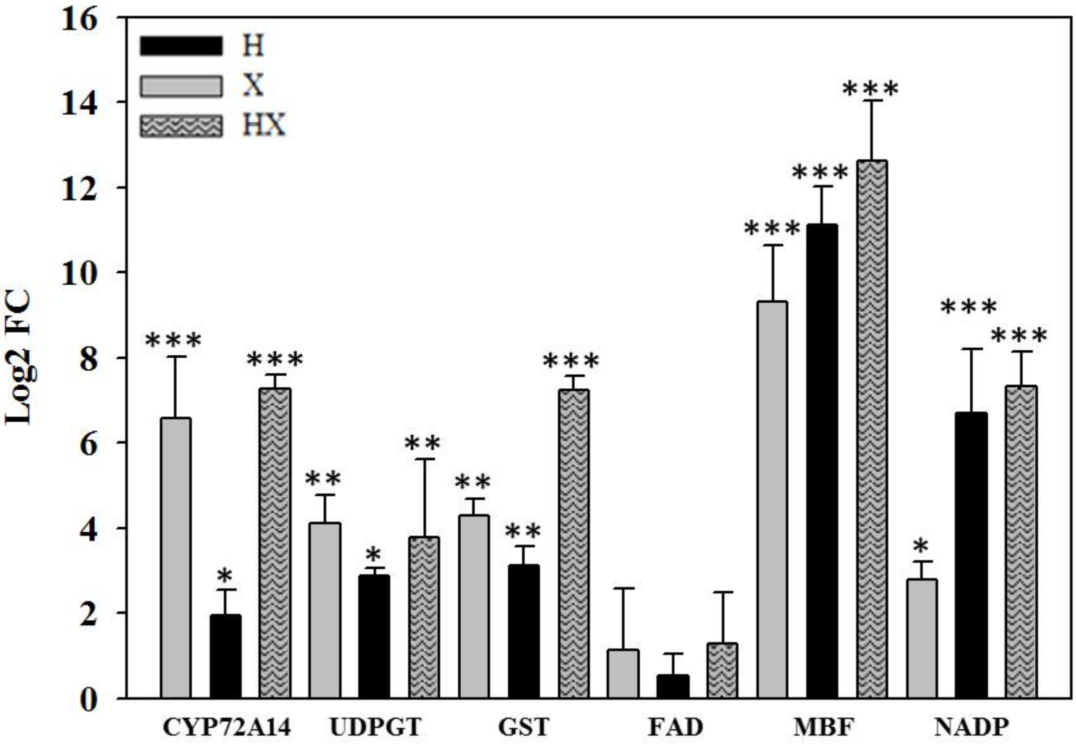

Figure S1. Relative expression of selected genes measured using qPCR under three treatments compared to control. Pinoxaden under control conditions (10/16°C) (X), high-temperature (H) (28/34°C) and treated plants under high-temperature (XH). Scale is mean of log2 fold-change values. qPCR values, representing mean ± SD (n = 3), were calculated and normalized using S-adenosylmethionine decarboxylase (Bradi5g14640.1) as internal control. Gene name according to their order of appearance (left tor right): CYP72A14 (Cytochrome P450, family 72, subfamily A, polypeptide 14, Bradi2g44150), UDPGT (UDP-glucosyl transferase 73D1, Bradi2g04720), GST (Glutathione S-transferase TAU 18, Bradi5g19400), FAD (FAD-dependent oxidoreductase family protein, Bradi4g35087), MBF (Multiprotein bridging factor 1C, bradi1g37080) and NADP (NAD(P)-linked oxidoreductase superfamily protein, Bradi3g48197). Differences between treatments to control were examined using Dunnett's t test,*, ** and *** indicate significance levels of $P \leq 0.05$, $P < 0.01$ and $P < 0.001$, respectively.
